# Learning from follow-up of student placements in a remote community: a small qualitative study highlights personal and workforce benefits and opportunities

**DOI:** 10.1186/s12909-019-1751-3

**Published:** 2019-09-04

**Authors:** Rosalie D. Thackrah, Sandra C. Thompson

**Affiliations:** Western Australian Centre for Rural Health, University of Western Australia, 35 Stirling Highway, Crawley, Western Australia 6009 Australia

**Keywords:** Rural career uptake, Clinical placements, Aboriginal health, Cultural immersion, Knowledge translation

## Abstract

**Background:**

The maldistribution of the Australian health workforce contributes to restricted accessibility and poorer health outcomes for rural and remote populations, especially Aboriginal and Torres Strait Islander Australians. Student exposure to rural and remote settings is a long-term strategy that aims to reduce workforce shortages by encouraging rural career uptake, with well-supervised, positive placement experiences associated with rural practice intentions. Furthermore, placements can build students’ cultural capabilities and foster interest in working with disadvantaged and underserved Aboriginal communities. However, little is known about the translation of rural practice intentions to career paths, and the factors influencing employment decision-making and application of clinical and cultural content to professional practice.

This in-depth study reports on the second stage of an investigation into the longer-term impact of remote placements. Stage One identified factors that contributed to students’ learning experiences and highlighted challenges encountered; Stage Two explored the impact on professional practice and employment decision-making amongst a subset of the original cohort.

**Methods:**

Of 12 interviews with participants who completed a remote placement in 2013/4 (Stage One), eight graduates were located four years later and seven were re-interviewed. Telephone interviews used a semi-structured schedule; each interview was recorded, transcribed and analysed for recurring themes and meanings.

**Results:**

At the time of interview, all participants were employed as health professionals and worked in Australia. The follow-up highlighted the enduring legacy of the student placement in terms of participants’ personal and professional growth. The majority were employed in rural settings; some were attracted by a rural lifestyle and employment opportunities while others were drawn by a desire to reduce rural health disparities. Regardless of setting, all actively applied clinical and cultural learnings acquired on placement to their professional practice. Rural job security, professional support and opportunities for professional development were all influences on continuing rural practice.

**Conclusions:**

Despite the challenges of qualitative longitudinal follow-up, the findings of this study provide valuable information, which can inform scaled-up investigations into the role of placements in developing an expanded, more stable and culturally respectful rural workforce.

## Background

Strategies to increase student exposure to the advantages of rural employment are essential to address workforce shortages and health disparities between rural and urban populations in Australia. Rural and remote exposure can also develop students’ cultural capabilities, essential for the provision of culturally safe health services to Aboriginal and Torres Strait Islander^1^ communities and foster socially responsive behaviours. In Australia, settlements beyond large metropolitan areas are defined as rural or “outer regional” [1] with the latter communities further differentiated based on size and distance from major towns [2]. “Remote” communities are characterised by highly dispersed small populations, which typically have restricted access to services and a higher proportion of Aboriginal Australians [1].

Since the establishment of University Departments of Rural Health in 1997 and Rural Clinical Schools in 2000, strategies to increase students’ rural exposure have focused upon rural-based medical education, training and support for rural health professionals, and the inclusion of rural and remote placements in largely urban-based medical and health science programs [3]. Evaluation of these strategies draws attention to employment locations of recent medical graduates [4, 5], and factors influencing placement satisfaction and rural practice intentions [6, 7]. Well-supervised and positive placement experiences have been identified as key to fostering rural practice intentions [7]. Student satisfaction and rural practice intentions are also linked to community engagement opportunities and placements of longer than 3 months duration [8], although less is known about the translation of practice intentions to career paths and factors influencing employment decision-making. Nationally based, longer-term follow-up studies of medical and health science graduates are recommended to address these issues [6, 7, 9].

A further area of interest, although less widely explored, relates to knowledge learned and insights gained by students while on placement and their application in subsequent employment in urban and rural settings. Rural and remote placements have the unique capacity to introduce students to the advantages of rural living and promote rural practice, while also exposing them to the health consequences of remoteness and the realities of rural-based social disadvantage. Furthermore, many rural settings have substantial Aboriginal populations: placements can provide valuable opportunities to build cultural respect and understanding, and observe the role of cultural protocols in health care delivery [10]. The impact of this exposure on students can be profound; humility, respect and a challenging of stereotypes have been observed as outcomes of sustained interactions [11].

Experiential and situated learning frameworks are frequently associated with cultural immersion placements [12]. These pedagogical approaches emphasise the strengths of learning “in situ”, in particular, the capacity for students to derive meaning and reflect upon their experiences and responses to placement settings [13]. Service learning models, which facilitate student engagement with communities through supervised service provision, can further enrich learning experiences and encourage the development of socially responsive behaviours [14]. Civic engagement can reveal a community’s strengthens and capacities to respond to social issues, particularly in geographically isolated and disadvantaged Aboriginal settings, and a positive experience may influence students’ future career paths.

Evaluation of strategies to foster rural practice intentions, including cultural immersion experiences, has occurred in parallel with an expanding body of literature on the recruitment and retention of health professionals in rural settings. Roots and Li’s meta-synthesis of factors influencing rural recruitment and retention among occupational therapists and physiotherapists, professions which are underrepresented in the rural workforce, highlighted professional support as significant to both recruitment of new graduates and their longer-term retention in rural settings [15]. Retention was also associated with “opportunities for career development and an understanding of the rural context prior to practising in a rural area” [15].

A recent scoping review of factors influencing rural retention among health professionals in Canada, the United States and Australia found that “place-based social processes” were related to decisions to remain or leave rural settings [16]. Social processes refer to interactions that lead to the development of social relationships; these may include community engagement opportunities, establishment of social networks and an attachment to place that arises from connectedness with the community. Emerging evidence suggests that the degree of social integration among rural health professionals is a contributing factor to retention rates with low levels of connectedness associated with a higher likelihood to leave [16]. The authors suggest that a better understanding of the social dimensions of retention, including those amenable to change, may enhance employment stability in rural health workforces [16].

The focus of this study was the follow-up of students who were involved in a pilot program in 2013 and spent 3–5 weeks of a semester-long rural placement in the remote town of Mt Magnet (Badimaya country^2^) in Western Australia. More specifically, the study aimed to explore the longer-term impact of the placement on professional practice and employment decision-making. The placement included strong input from the Mt Magnet Aboriginal community and provided opportunities for students from a range of health science professions to work with children at the local school, to connect with community organisations and to participate in local events [12]. Academic staff at the Western Australian Centre for Rural Health (WACRH) based in Geraldton in the state’s Mid-West conducted student supervision both ‘in situ’ and remotely. The local Aboriginal staff member employed by WACRH, acted as cultural mentor and played a vital role in providing social support and guidance on cultural and community issues. The placement aimed to facilitate interprofessional learning, develop cultural capabilities and encourage rural practice through positive exposure. Previous research has shown that students select such placements for a number reasons, including interest in rural and/or Aboriginal settings, assistance with accommodation and travel, and for any potential “edge” the experience may provide in securing employment [12].

Small, in-depth qualitative studies have drawn attention to the personal and professional implications of cultural immersion experiences [10]. Few, however, have considered whether and how recent graduates have opportunities to apply clinical and cultural learnings acquired on these placements in workplaces after graduation, if they are supported in this process, and the factors that influence employment choices in the early years of their careers. In-depth longitudinal studies can contribute to a better understanding of the impact of knowledge acquired, early career development and workplace environments, and the decision-making process around employment choices and locations. While small in scale, this follow-up study builds on earlier findings and highlights the potential contribution that rich, longitudinal data can make to our understanding of students’ placement experiences and the impact of these experiences on practice and employment choices.

## Methods

The study was conducted in two stages. The first stage identified factors that contributed to positive learning experiences by 12 participants on a remote, interprofessional clinical placement and also highlighted challenges encountered. Stage Two which is described in this paper, drew upon these findings to explore the longer-term impact of the placement, especially with reference to rural employment and the application of knowledge acquired on placement to professional practice.

The setting and preparatory support for students in the program is presented in full in the paper describing Stage One [12], and only in brief here. Mt Magnet lies 342 km east of Geraldton [17] and was selected as a suitable location for placements due to long-standing relationships between WACRH staff and local Aboriginal community members. It has also been identified by WACRH as a high needs setting where students could make a contribution to service delivery while simultaneously developing clinical skills and cultural capabilities. Purchase of a house in Mt Magnet to provide accommodation for visiting WACRH staff and students made development of clinical and immersive placements possible [12]. At the time of the first stage of the study Mt Magnet had a population of 532 people; 43.5% (230) identified as Aboriginal Australians [18].

In-depth interviews were used in the first stage of the study in 2014–5; all 12 participants had agreed to be contacted for the second stage and were sought for follow-up interview. Various methods were used to locate participants including emails, phone calls, text messages and social media. Only eight of the 12 participants could be located three years later and all agreed to a follow-up interview, although one was subsequently unavailable. Participants were deemed to be uncontactable following at least three unsuccessful attempts to make contact. All the follow-up interviews were conducted by telephone due to the constraints of work and the widely dispersed settings of the participants. The same interviewer [RDT] conducted all first and second stage interviewers.

A semi-structured interview schedule developed by the researchers guided the interviews, which ranged in length from 40 to 65 min. Participant Information and Consent forms were provided in advance of interviews, which did not proceed until signed or verbal recorded consent was obtained. The interview focused on each participant’s experiences and reflections on their career, including the impact of their “in situ” learning from their Mt Magnet placement. All recordings were transcribed by a professional transcription service, allocated a code to safeguard confidentiality and erased by the service after eight days.

Standard qualitative research techniques were employed to analyse data. These included multiple readings of the transcripts, annotations and bracketing of quotations and examples, coding and categorisation of emerging themes, discussion within the research team, and identification of final themes and associated patterns of meaning [19]. Immersion in the data is a hallmark of qualitative research and supports rigour in data analysis and reliability in the findings. Ethics approval for the second stage of the research was obtained from the Office of Human Ethics at the University of Western Australia.

## Results

### Participant characteristics

The participants’ ages ranged from 25 to 33 years; all were female and Australian-born. Three were raised in metropolitan settings while four grew up in small rural or larger regional towns. At the time of interview all were employed and five of the seven were living in regional or rural locations including Northampton and Newman in Western Australia, Lismore (New South Wales), Cairns (Queensland) and Alice Springs (Northern Territory). Of the remaining two, one had just completed three years in Kalgoorlie, 590 km east of Perth and returned to the city several weeks before the interview to take up a new position; the other was Perth-based, working in a role that required regular country travel. All worked in areas related to their qualifications; three were occupational therapists, three speech pathologists and one was a generalist health science graduate. Four participants worked in the public sector; three in health and one in education, while the remaining participants worked for charity-based, non-profit organisations (two) and a private speech pathology clinic (one). Two health disciplines from the original cohort were not represented in the Stage Two interviews: social work (two participants) and exercise physiology (one participant).

### Employment histories

#### Labour market conditions

All participants graduated in 2014/15 and most acknowledged the tight labour market conditions that prevailed at that time, particularly in Western Australia. Anxieties about securing and maintaining a position, which were frequently expressed in the first stage of the study, were reflected again in the second stage interviews: casual short-term contract positions were commonly offered and accepted as a stepping stone to a more secure role. One participant took nearly two years to obtain a position as an occupational therapist in a regional town, only to find that three-year contacts had been reduced to 12 months.

All participants commented on the high turnover of staff due to short–term contracts and last minute renewal of contracts was a source of considerable stress for some. Despite this it was suggested that others used short-term contracts to their advantage, and that early career professionals expected insecurity in the workplace so were not averse to relocating for employment: *“Everyone who comes to Cairns loves it, but it is like they have already made up their mind to go home after a year. It is like a working holiday”*. However, a desire for permanency was identified as important by those who had partnered and wanted to start a family: *“I hope to still be working at the hospital in two years’ time but I do want permanency … I’d love to stay (where I am) but I’ll leave, only because of the permanency issue; this is a contract position”.*

#### Rewards and challenges

Participants identified many rewards in their current roles. Being surrounded by supportive colleagues was noted by all as essential to their wellbeing and capacity to fulfil their duties as a new recruit. Most also referred to enjoyment derived from interacting and developing strong relationships with their clients and their families. Others drew attention to the nature of the position, its flexibility, the independence offered and its strong community focus. *“I realised I would rather be out here doing things on the ground*... *working in an organisation whose main business is community development and sustainability.”* Regular country travel was the highlight for the city-based participant who took up her position soon after graduation.

Challenges identified were diverse: heavy and complex caseloads, lack of opportunities to specialise, making friends and losing them due to high staff turnover, funding issues and job insecurity. Work related challenges were largely ameliorated through collegial support. All participants drew attention to the role of supportive colleagues and management teams in providing mentorship and advice on difficult cases. *“I found the work very draining [at the beginning] and had trouble switching off after work*. *.*. *I had a good colleague who kind of acted in a mentor role and it was vital to be able to talk to her”.* Another noted that *“having the opportunity to bounce things off my colleagues and discuss difficult circumstances with my seniors [helped me through difficult days]*. *.*. *the senior support and collegial support has been amazing”.* Participants’ comments suggested that the small team environments typical of country practice were conducive to enhanced support and mentoring of new graduates.

#### Impact of remote placement on securing employment

With only one exception, participants indicated that the remote placement at Mt Magnet, which they completed during their program gave them “a foot in the door” when it came securing their first professional position. One participant commented that she

*“definitely brought it up in interviews . . . it shows that you have the skills they are looking for, initiative and independence. They often worry when they send you to remote communities because you are pretty isolated . . . they ask if you are strong enough and have the right supports. Saying I’ve been to Mt Magnet and am confident I would cope living out there definitely helped.”*

Another commented that she was advised her placement experience working in rural locations with different populations had placed her ahead of other applicants, even though it was not an essential job criterion. Participants also noted that employers sought evidence of cultural capabilities; most considered they were well placed to draw upon their experiences interacting with Aboriginal community members to demonstrate these attributes. While most participants emphasised the importance of professional experience in career advancement, five regarded the placement as also playing a role, with two of these identifying it as directly responsible for their career advancement.

### Knowledge translation: placement to practice

#### Working in Aboriginal contexts

All participants had professional roles that involved working with Aboriginal clients although the number and nature of interactions varied. All agreed that the remote placement had equipped them to work in Aboriginal contexts although one felt that her learning was impeded due to a lack confidence at the time. Another considered that her initial exposure to Aboriginal communities at Mt Magnet had “sowed the seed” that led to an on-going commitment to work in Aboriginal health and education. She noted that the placement prepared her well to work in Aboriginal communities, but she had since realised how inherently diverse these communities are:

*“I wasn’t exposed to working with Martu specific people [on placement] . . . I’m still learning the Martu way . . . here in Newman I’m learning about cultural ways that are similar and different. The biggest thing I’ve noticed here is how strong language is. I was so surprised that mums were talking to their little kids in language”.*

One participant commented on the authentic, uncensored nature of the Mt Magnet experience:

*“ What I learned and the experiences I had at Mt Magnet . . . probably shaped the way I am as an OT [occupational therapist] . . . that experience was very real, you couldn’t push it away or hide it under the carpet . . .[we were exposed to] families living in such difficult circumstances”.*

Other participants also expressed this sentiment:

*“I still remember things I learned out there . . . especially when things aren’t working out as much as you try [and you’re blaming yourself] . . . then you remember ‘oh, maybe they didn’t have breakfast this morning, or maybe they were sharing a bed with others and didn’t get much sleep’ . . . I am much more understanding because of that experience”.*

*“I am more aware of the complex family lives and circumstances [as a result of the placement]*. *.*. *and much less judgemental. I felt better prepared than my colleagues”.*

All participants considered that they had been exposed to adequate content in their programs, placements and workplaces to build their cultural capabilities and apply this knowledge in clinical and community practice.

#### Rapport and flexibility

Participants provided numerous and detailed examples of how they applied their remote placement learnings to professional practice. An ability to relate to and build rapport with Aboriginal community members was identified by all as a capability acquired on placement and refined in professional practice as they had more interactions with Aboriginal community members. A speech therapist identified flexibility and humility as important attributes acquired on placement.

*“I would say most importantly my attitude towards working with Aboriginal and Torres Strait Islanders is something that I learned most out there [at Mt Magnet] . . . I think being just more relaxed and knowing that it’s okay if it doesn’t happen the way I expect it to . . . also being able to approach people in a more humble kind of way. Some people want to see you as the real “professional”, the walk, the talk, but for others, it makes them feel uncomfortable . . . [unless they’re comfortable] they just misinterpret what you are saying or don’t engage with you again”.*

For one occupational therapist working in a regional hospital, cultural understandings and the importance of language were considered essential to establishing rapport and trust with Aboriginal clients. Flexibility and the need to “adapt assessments to make them relevant to the clientele” were closely associated with this approach.

*“About 80% of our hospital in-patients are Aboriginal . . . we use less formal assessments and work closely with our ALOs [Aboriginal Liaison Officers] and get a lot of information that way. I avoid almost all formal assessments because they are not relevant, they are not standardised to any of the people here or their circumstances at home. My assessments are mostly functional reviews . . . it is harder in the hospital environment because we don’t have campfires to sit around and see if they can make a billy. But we work within our kitchen environment . . . we would never sit down and do a paper and pen task. We use translators and ALOs for anyone who cannot speak English very well or if we aren’t sure if they understand what’s going on.*

*Personally, I’ve being learning Pitjantjatjara* 3 *for 2 years . . . I feel it helps me build rapport with people in a big way. I think it is one of the big things because they have been spoken at so much here from being in the RFDS [Royal Flying Doctor Service] to ED [Emergency Department] to the wards, all in English, probably all very fast medical language . . . so for someone to come in and say “Hey! How’s it going?” in* ***their*** *language, they really like it”*

#### Interprofessional learning (IPL)

The remote placement completed by participants had a strong IPL component and all viewed the interprofessional exchange opportunities provided as valuable in subsequent workplace settings. *“It helped me gain more respect for the other professions, which I should have had anyway*. *.*. *and it helped me understand the referral process”.* Another noted that IPL was not widely used in her current setting, as older people in the workforce were less familiar with this model of collaboration. *“It is one of those things I have to advocate for because it is a newer model of working, and maybe if I didn’t learn it out there [on placement] it wouldn’t be done at all. It’s definitely the newer people to the profession that do it.”* One participant suggested that working together as a united team achieves better outcomes for clients and another commented on interprofessional collaboration as an important underpinning within the National Disability Insurance Scheme (NDIS) environment where she is employed.

### Rural intentions

Participants were asked whether the remote placement had influenced their thinking about rural employment, where they might be in two and five years’ time, and their thoughts on attracting health professionals to rural and regional areas. All commented that their placement had influenced their decision to work rurally, either immediately upon graduation or into the future. Three participants had a pre-existing interest in rural employment and noted that the placement reinforced their enthusiasm for the rural lifestyle, while the remainder suggested that their rural intentions arose out of positive placement experiences and better job opportunities post-graduation. Rural intentions were not differentiated according to where participants were raised; those from both urban and rural backgrounds considered the placement influenced their immediate and longer-term employment decision-making. The city-based participant, who regularly travels to the country for work, expects to live rurally within five years. As one participant who was born and grew up in the country noted:

*“I wanted to move away from the country but did a couple of placements including Broken Hill, Geraldton and Mt Magnet, and they were a lot of fun. It’s a good way to explore new places. Those placements have definitely made me more interested [in going to remote places] rather than staying on the coast where I always thought I’d be after leaving home.”*

The immediate and longer term plans of participants were influenced by both personal and professional factors. Given their age profile, most referred to “settling down” and starting a family within the next five years; this focus again surfaced concerns around employment security. Two of the seven participants had considered completing a Diploma of Education.

*“If I had my Dip Ed and my OT [occupational therapy] degree I would have more prospects for a permanent job . . . my partner is a farmer and farming is such a big gamble . . . it is quite important to me to have a secure job, just for future planning”.*

Several participants identified professional limitations associated with remaining in the country, including one who had recently moved to the city to pursue specialist training. *“Professionally and clinically my particular interests make it a bit difficult to work in regional areas*. *.*. *I loved working in [the country] and that’s why I stayed so long, but the thing that really drew me home last month [to the city] was that I wanted to gain more experience in a very specific area*. *.*. *you don’t get the opportunity to do that in rural areas”.* The majority, however, expected to remain in rural settings; four of the seven participants identified a specific intention to work in Aboriginal settings into the future. “*I find positions with Aboriginal communities the most challenging and they are the ones where service delivery is probably least effective*. *.*. *I guess I think it could be done so much better*. *.*. *I just want it to be done better and contribute to this”.*

All participants referred to increased opportunities for rural placements and the impact of rural exposure as factors in building the rural health care workforce. *“I’ve had quite a few friends from the city who did not want a rural placement but they got placed there anyway and then ended up working rurally forever, or at least up until now. I think exposure is probably the key”.* Some noted that those more likely to stay in rural settings were active in the community and had well-established support networks. For another it was “*well, what has kept me? I met a guy here!*” Higher pay, flexibility in work, and faster career advancement were also identified as draw cards which should be promoted to students and graduates. Lastly, a link was made between positive and well-supported rural placements in their undergraduate program and heightened interest in rural practice, although the challenges in generating initial interest among city-based students to “go rural” were acknowledged.

## Discussion

The maldistribution of the Australian health workforce contributes to poorer health outcomes and restricted service accessibility for rural and remote populations [20]. Government investment in rural clinical placements for medical, dental, nursing and allied health students is a strategic response to the rural health professional deficit. Given that positive placement experiences can influence rural practice intentions [7, 8], on-going funding is vital to provide opportunities for rural exposure during training. However, it is not guaranteed that end of placement intentions will be translated to workforce outcomes.

This study of a small cohort of students provides insight into employment decision-making and practice implications of placement learnings through longitudinal follow-up. Previous documentation noted that an important component of students’ learning arose from their immersion in a small, remote community with many Aboriginal residents. In this setting, students observed the impact of isolation and social disadvantage on health outcomes at close range, and also witnessed the community’s strengths and resolve in responding to serious social issues [12].

The importance and potentially transformative impact of such placements in Aboriginal settings has been described by a number of authors, and most recently been reported in a systematic review [10]. The review also identified the limitations of such studies: the lack of an independent measure to assess skill in working with Aboriginal people; whether the knowledge acquired on placement prepares students for encounters with Aboriginal people in mainstream settings; and whether a rural health placement influences students’ decision-making to work rurally or in Aboriginal health [10]. In medicine, studies reporting on the longer-term impact of rural or Aboriginal field placements on early career location in terms of rural career uptake have been encouraging [4, 5, 21]. However, less is known about the influence of placements on rural career uptake and retention among non-medical health professionals. This is despite government funding for placement programs and the need for evidence of their effectiveness in improving health workforce outcomes.

In-depth follow-up studies are inevitably restricted to small numbers. This study highlights the challenges of follow-up in longitudinal studies among a group of young, female, mobile, recently graduated health professionals whose location and contact details may change. Although the current study was foreshadowed with students at the time of their original placement and previous participation, graduates move on to new and busy lives while establishing their careers. Longitudinal studies must find a balance between pursuing potential study participants who are not readily contacted, while not harassing any who do not respond (by choice or otherwise). Of note, eight of 12 former participants contacted agreed to be interviewed, although one subsequently withdrew for logistical and scheduling reasons. The findings indicate respondents’ genuine interest in contributing to a better understanding of rural health workforce issues among new graduates, and all agreed to be contacted again in three years’ time.

Despite the challenges of follow-up, the findings highlight the enduring legacy of the remote placement: participants recognised that the placement expedited both their personal and professional growth. It had often opened their eyes to the social underpinnings of poor health as reflected in the challenges faced by Aboriginal people, and all provided examples of how they had applied their cultural and clinical learnings in practice. Importantly, many remained committed to working in rural or remote health and contributing to efforts to reduce health disparities in Aboriginal communities; shortfalls in service delivery were identified and a desire to address these expressed. Familiarity with the context of rural practice is a contributing factor to retention [15] and the placement exposure was identified by participants as better preparing them for working and living in a rural setting. However, there is a need to keep a big picture view, as it was rural communities distant from the placement site that were the beneficiaries of these students’ experience. Their experience was reflected in interest in rural health as well as sensitivity and expertise as health professionals. Of note, secure employment in their chosen profession was a paramount consideration in their career planning regardless of an individual’s desire to contribute to improved health and wellbeing for rural and Aboriginal communities.

The findings reinforce the importance of a workforce strategy and funding for nursing and allied health students that goes beyond student clinical placements. What kept most of the participants working in rural areas was finding employment. However, a repeated theme was of short-term contracts and little job security making it harder to “settle” and to sustain being a member of the community and rural health workforce. This reflects the much larger, contemporary issue of short-term contracts, but it undoubtedly undermines the development of a stable rural workforce. Retention of rural health care providers has been linked to professional support, career development opportunities and the establishment of social connections in rural communities [15, 16], all more likely to occur in an environment that offers job security.

The Australian government has now funded rural clinical training hubs to keep rurally trained new medical graduates in rural areas while they develop their skills and knowledge post-graduation [22]. Similar support for graduate allied health professionals in rural areas has not yet occurred, but as highlighted by the literature on retention and by this research, there also is a need for systematic, on-going professional support for other new and existing health science graduates if they are to be retained long-term in the rural health workforce.

### Limitations

Two-thirds of participants in the first stage of the study were located three years later, with seven of the eight re-interviewed. This attrition rate highlights the challenges of longitudinal studies. All participants re-interviewed were female; reflecting the gender differential of the original cohort and the included allied health professional groups. The potential for bias exists where rural placement experiences are linked to rural career uptake; if placements are voluntary, a pre-existing interest in rural practice may lead to self-selection. Notwithstanding this, the rich qualitative data gathered provides encouraging evidence of the longer-term impact of rural placements on subsequent clinical and community practice, and rural career uptake.

## Conclusions

Well-supervised rural and remote clinical placements introduce students to the advantages of rural living while simultaneously exposing them to the health consequences of rural-based social disadvantage. For some participants, student placement exposure “sowed the seed” to commit to working rurally and contribute to reducing health inequities, especially within Aboriginal communities. For others, a rural lifestyle or enhanced employment opportunities were drawcards after positive placement experiences.

Overall, the findings reveal an enduring legacy of the placement in terms of participants’ personal and professional growth. Most were employed in a rural setting and actively applied knowledge learned on placement to clinical practice. Their long-term retention in a rural area was likely to be affected by insecure employment and limited opportunities to specialise, with professional support being identified as essential to workplace satisfaction.

Despite inherent difficulties, scaled–up, qualitative, longitudinal studies of allied health professionals are required to explore employment decision-making and the contribution of rural placements to an expanded, more stable and culturally respectful rural workforce.

## Data Availability

Data sharing is not applicable to this article as no datasets were generated or analysed during the current study.

## Abbreviations

ALO: Aboriginal Liaison Officer
Dip Ed: Diploma of Education
ED: Emergency Department
IPL: Interprofessional Learning
NDIS: National Disability Insurance Scheme
OT: Occupational Therapy
RFDS: Royal Flying Doctor Service
WACRH: Western Australian Centre for Rural Health

## Footnotes

1 The Indigenous peoples of Australia include Aboriginal people and Torres Strait Islanders. In Western Australia, Torres Strait Islanders represent a very small proportion of Indigenous Australians; in this setting there is a preference for the use of the term Aboriginal over terms such as Indigenous or abbreviations such as ATSI. For the purpose of this paper, set in remote Western Australia, we respectfully use the term Aboriginal to refer to the Aboriginal and Torres Strait Islander peoples of Australia. The term Indigenous appears in this paper when reference is made to literature using the term or when it is used in quotations.

2 Badimaya country is located within Yamatji country, which encompasses the Murchison and Gascoyne regions of Western Australia.

3 Pitjantjatjara is a language of the Western Desert Language Group and is spoken by Pitjantjatjara people of Central Australia.
